# Explainable machine-learning-based predictions of blood lead levels and school drinking water contamination among children: a case study in Washington DC

**DOI:** 10.1038/s41598-025-24213-2

**Published:** 2025-11-18

**Authors:** Dylan Darling, Yogesh Bhattarai, Sara Kamanmalek, Rocky Talchabhadel, Sanjib Sharma

**Affiliations:** 1 Department of Civil and Environmental Engineering, Howard University, Washington, D.C, 20060 USA; 2 Department of Civil and Environmental Engineering, Jackson State University, Jackson, Mississippi 39217 USA

**Keywords:** Urban water infrastructure, Boil water advisory, Lead contamination, Risk prediction, Machine learning, Engineering, Environmental sciences, Natural hazards

## Abstract

Water quality degradation poses significant risks to human health, ecosystem, and community. Many cities continue to rely on outdated pipes and water distribution networks that are highly susceptible to leaks, corrosion, and lead contamination. The processes driving lead contamination are evolving with aging infrastructure and changing environment, and there remains a critical challenge for predicting the associated risk. The key objective of this study is to improve the understanding and prediction of blood lead levels and school drinking water contamination among children using explainable machine learning. Focusing on Washington, District of Columbia, where lead exposure remains a persistent concern, we develop and evaluate random forest, adaptive boosting, and gradient boosting models using environmental, topographic, socioeconomic, and infrastructure features as predictive inputs. We then apply Shapley additive explanations to quantify the relative influence of each variable on model outcomes. Results demonstrate strong discriminative ability across all models, with area under the receiver operating characteristic curve ranging from 0.90 to 0.95. Ensemble-based approaches consistently outperform logistic regression, achieving higher accuracy, precision, recall, and F1-scores, along with narrower confidence intervals. Over 11% of the city lies into very high-risk zone, and 13% is classified as a high-risk zone. In particular, Wards 1, 4, and 6 are among the most impacted areas, exhibiting high concentrations of lead service lines and elevated predicted contamination risk. City-wide predictions are primarily driven by lead pipe density and social vulnerability, while school-level risks are more strongly influenced by water infrastructure characteristics, including device type and building age. These findings offer critical insights for guiding targeted interventions such as lead service line replacements, prioritization of high-risk schools, and resource allocation to vulnerable neighborhoods.

## Introduction

Over two billion people live in water-stressed regions with limited water supply and poor drinking water quality^1^. Many cities rely on outdated and aging water infrastructures, such as pipes, treatment facilities, and distribution networks that are susceptible to leaks, corrosion, and lead contamination^2^. Water quality degradation has devastating impacts on human health, ecosystem and community. In particular, lead is a neurotoxin that can cause severe health risks, particularly for young children, pregnant women and elderly people. Globally, approximately 800 million children have blood lead levels above 5 $$\upmu$$g/dL, a threshold deemed harmful by the World Health Organization^3^. In the United States (US), lead-contaminated drinking water systems remain a persistent issue^4^, with annual costs exceeding $50 billion due to lost productivity and healthcare expenses^5^. Half of the US population, more than 170 million people, were exposed to harmful lead levels in early childhood^6^. Although lead was banned from new plumbing systems in the US in 1986, a significant portion of the nation’s drinking water infrastructure was built before this regulation^7^. Lead pipes are widespread across the country, and large metropolitan cities such as Chicago, Cleveland, New York, Washington District of Columbia (DC), and Detroit experience more severe^8^. Marginalized and low-income communities disproportionately bear the burden of lead contamination^4,9^. Although regulatory measures such as the Lead and Copper Rule^10^ aim to limit lead levels in drinking water, millions of people remain at risk.

Lead contamination remains a persistent public health concern in Washington, DC^11^. In the early 2000 s, the city faced a major crisis when a change in water treatment methods triggered a spike in lead levels. In 2001, the DC Water and Sewer Authority switched from chlorine to chloramine to reduce harmful byproducts. However, this shift inadvertently increased the water’s corrosivity, causing lead to leach from aging pipes into the water supply^12^. As a result, some houses exceeded the federal action lead level of 15 parts per billion (ppb). Given the high prevalence of homes built before 1978, DC faces a high-risk area of lead hazards. Over 63% of owner-occupied and 34% of renter-occupied units were built before 1950^13^. DC also exceeds all other states in the proportion of housing (34%) built in 1939 or earlier, when lead-based paint was likely used in 90% of homes. Although initiatives like Lead-Free DC have accelerated the replacement of lead service lines, more than 40,000 lead service lines are still in place^7,14^. Despite its vulnerability and historical significance in national lead crises, DC has received limited attention in prior lead risk modeling studies. Predictive modeling of lead contamination risk is critical to support targeted interventions, inform long-term infrastructure investments, and mitigate the severe consequences of water quality deterioration.

Researchers and practitioners have focused on developing accurate, real-time lead contamination risk prediction models to help decision-makers identify potential contamination areas and proactively mitigate their impacts. Several previous studies^15,16^ have employed geostatistical approaches to understand and identify lead risk in drinking water systems. Statistical models often rely on linearity and distributional assumptions, which limit their ability to capture complex, spatially diverse lead risk factors. Recent advances in machine learning provide a powerful alternative to traditional modeling approaches for predicting lead contamination risk. Machine learning algorithms can process large and diverse datasets to identify complex, non-linear relationships among multiple risk factors, enabling more accurate and scalable predictions^17,18^. Given that lead exposure remains a high-stakes public health concern, especially for vulnerable populations, there is a pressing need for more robust and interpretable predictive approaches. However, the black box nature of many machine learning models can obscure the reasoning behind predictions, making it difficult for stakeholders to trust the results or translate them into action^19^. This underscores the need for explainable machine learning approaches that improve transparency, identify key risk factors, and support informed and accountable decision-making^20^.

Previous studies have shown important applications of machine learning in improving various aspects of lead risk assessment, including simulating lead concentrations in water^21^, identifying houses with high lead tap water concentrations^22^, predicting the pipe materials house-by-house throughout a water system^23^ and classifying water lead levels in private drinking water systems^24^. Recently, Huynh et al. 2024^25^ used a machine learning model to estimate childhood lead exposure from drinking water in Chicago, Illinois. Similarly, Mulhern et al. (2022)^26^ employs machine-learned Bayesian networks to assess the relationship between children’s blood lead levels and drinking water systems in North Carolina. Identifying lead contamination risk at the city level helps target neighborhoods with the highest contamination risk, enabling policymakers to prioritize infrastructure upgrades, lead service line replacements, and community outreach efforts.

While previous studies focus mostly on residential or regional water systems, very few have applied machine learning to assess the unique vulnerabilities of school drinking water systems^27^, where children who are particularly vulnerable to lead exposure can face higher risk. Schools present distinct and often overlooked challenges, including aging plumbing infrastructure, intermittent water use, prolonged water stagnation, and variation in the construction year of school buildings. These characteristics can significantly influence lead release. However, they are typically not captured in broader regional analyses, which often generalize risk across larger spatial units and overlook fine-scale variations in usage patterns and building conditions. As a result, regional frameworks may not detect site-specific risks that are critical for protecting school populations. Developing an integrated framework that combines neighborhood-scale and school-specific assessments can provide more nuanced and comprehensive insights into lead contamination risk, capturing both systemic patterns and localized vulnerabilities essential for effective intervention and equitable resource allocation.

The key objective of this study is to improve the understanding and prediction of blood lead levels and school drinking water contamination among children by integrating hazard, exposure, and vulnerability factors. To achieve this, we train and evaluate logistic regression, random forest, adaptive boosting, extreme gradient boosting, and light gradient boosting algorithms using hydrologic, environmental, topographic, socioeconomic, and infrastructure features as predictor variables. We then apply SHapley Additive exPlanations (SHAP) to interpret the models, quantify the relative importance of individual predictors, uncover interactions among variables, and elucidate the reasoning behind specific predictions. This explainable machine learning framework enables transparent, data-driven insights that can inform targeted interventions to reduce lead exposure in schools and high-risk communities. Key contributions of this study include: (i) integrating city-wide environmental, infrastructural, and socioeconomic data with health surveillance records to develop a comprehensive framework for predicting the geographic likelihood of elevated child blood lead levels; (ii) employing a school-level model that leverages direct water measurements to predict lead contamination in drinking water fixtures, providing an environmental contamination framework for infrastructure management; (iii) applying explainable machine learning algorithms to identify the most influential predictor of elevated blood lead level and school-level water contamination; and (iv) conducting a focused analysis on DC–a city with some of the nation’s oldest water infrastructure, a high density of older housing, and a well-documented history of lead contamination.

In this study, we use elevated blood lead levels in children as a proxy for geographic likelihood of city-wide lead exposure. We acknowledge that blood lead levels reflect multiple exposure pathways, including lead-based paint, contaminated soil, and dietary sources, and is not solely determined by drinking water. Nevertheless, elevated blood lead levels remains the most comprehensive and systematically collected health outcome available at the city scale, providing a socio-epidemiological perspective on child lead exposure risk. Historical data in Washington, DC indicate that aging water infrastructure has been a significant contributor to elevated blood lead levels^7,11^, supporting the relevance of this proxy. Importantly, the city-level model is explicitly framed as a socio-epidemiological prediction task and is distinct from the school-level model, which directly predicts water lead contamination using measured water samples.

## Methods and materials

### Study area

**Fig. 1:**
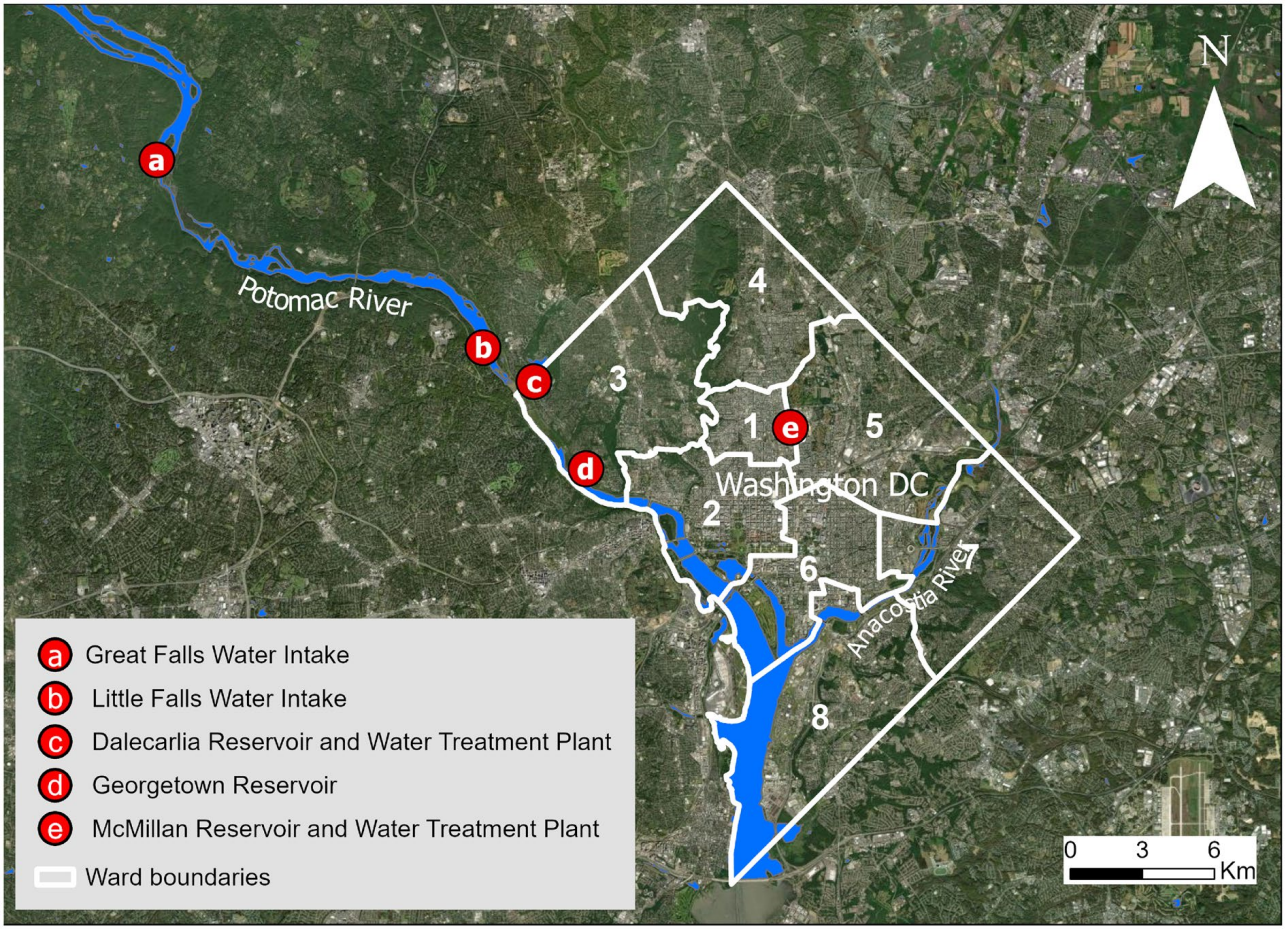
DC Water Delivery System, including intake, water treatment plant and reservoir. Basemap is obtained from ESRI worldmap V2^31^. Map created using ArcGIS Pro^32^.

The primary water supply for DC comes from the Potomac River, which serves more than 700,000 residents^28^. The Potomac River originates in the Appalachian Mountains and flows into the Chesapeake Bay, the largest estuary in the US (Fig. 1). As the river moves downstream, water is drawn from several intake points, where it is stored and treated for drinking. Water treatment facilities, such as the Washington Aqueduct and McMillan Reservoir, process the water from these intake points to ensure it meets safety standards for municipal use^29^. The treatment process typically begins with primary filtration to remove pollutants and sediments, followed by secondary and tertiary treatments to eliminate biological and chemical contaminants^30^. Once treated, the water is distributed throughout the city under the supervision of the DC Water and Sewer Authority. DC Water manages an extensive network of over 1,300 miles of underground pipes and continuously monitors the system to maintain water quality^30^.

**Fig. 2:**
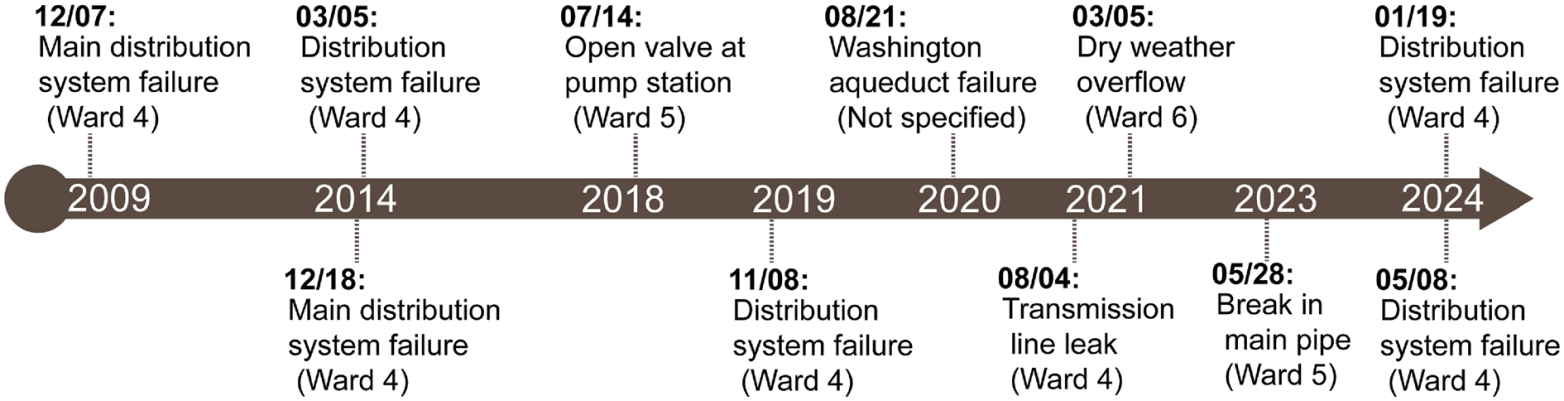
Timeline for boil water advisory issued in Washington D.C^36^.

Several boil-water advisory has been issued in recent decades (Fig. 2)^33^. These advisories are mainly associated with failures in water infrastructure systems, including pressure losses, pipe breaks and sewage system disruptions^34^. Pressure fluctuations and system flushing can disturb lead-bearing pipes and fittings, which can mobilize lead particles and increase lead concentrations in drinking water^35^. These hydraulic disturbances may also disrupt corrosion control measures. In addition, fluctuations in water treatment processes, particularly emergency responses to contamination events, can alter water chemistry and compromise corrosion control measures.

Public schools in DC are distributed across all wards, with higher concentrations in densely populated areas. Many are located in clusters near Rock Creek Park, Capitol Hill, and neighborhoods east of the Anacostia River (Fig. 1). The school infrastructure is highly heterogeneous, with buildings ranging in construction from as early as 1868 to more recent facilities, and a substantial proportion built prior 1970^37^. Major modernization initiatives between 1998 and 2012, along with ongoing renovations, have introduced variability in building conditions and design standards. Water fixtures within schools also reflect this diversity, ranging from legacy plumbing and traditional drinking fountains to newer bottle-filling stations and filtered sinks that comply with updated safety requirements.

### Data and feature descriptions

#### Lead contamination risk data

For city-wide analysis, we use the Children’s Lead Screening Report (2016–2019) from the Department of Energy and Environment (DOEE)^11^ to identify cases of elevated blood lead levels as an indicator of lead contamination risk. Elevated blood lead level is one single blood lead test (capillary or venous) at or above the blood lead reference value of 5 micrograms per deciliter ($\upmu$g/dL), as established by the Centers for Disease Control and Prevention. Using data from 2016 to 2019, DOEE identified clusters of elevated blood lead levels in children below six years of age through GIS-based mapping techniques. We integrate this cluster with hydrologic, environmental, topographic, socioeconomic, and infrastructure features to predict city-wide lead risk.

We use water test data from public schools in DC, collected by the Department of General Services (DGS)^38^, as an indicator of lead contamination. The tests from DGS covers various water sources, including bottle-filling stations, food preparation sinks, health suite sinks, classroom sinks, and staff lounge sinks. Each test includes two measurements: Draw 1, taken after water has been stagnant in the plumbing for 8 to 18 hours, and Draw 2, collected after flushing the pipes to remove stagnant water. For our analysis, we use lead concentration from Draw 1 as the target variable and apply a binary classification threshold of 1 ppb. We select 1 ppb threshold because no amount of lead in drinking water is considered safe, and it reflects the District’s health-protective target^39^. After removing samples with missing values, the final dataset includes 8,041 samples for each class.

#### Lead contamination conditioning factors

We use a range of hydrologic, environmental, topographic, socioeconomic, and infrastructure features for predicting lead contamination risk^6,23,25,26^. For the citywide predictive model, these features include house construction year, land surface temperature, distance to the nearest school, lead pipe density, distance to non-lead pipes, population density, percentage of population under 18 years of age, Social Vulnerability Index (SVI), distance from the water distribution system, distance to the nearest stream, and distance to combined stormwater outfalls. For the school-based model, we replace the house construction year with the school construction year, add the device type from which the water sample is collected, and exclude the distance to school, as all samples are within school facilities. These features were selected based on prior studies that identified them as key factors influencing lead contamination risk^21,25,27^.

The year of construction of the house and schools are classified into two classes: built before 1986 and built after 1987, to reflect the regulatory ban on lead pipes. This feature is treated as a binary categorical variable in machine learning models.Land surface temperature captures the exchange of energy and moisture between the Earth’s surface and the atmosphere^40^. Elevated temperatures can increase the solubility of lead in water, exacerbating corrosion and raising contamination risks. We use average land surface temperature data from Open Data DC, covering the years 2014 to 2018. These values are derived from Landsat 8 Level 1 imagery^41^.We compute Euclidean distances from each spatial grid cell to key infrastructures, including the nearest school, nearest non-lead service line, nearest water distribution system, and nearest combined stormwater outfall. These distances help quantify the spatial proximity of vulnerable populations and infrastructure conditions potentially influencing lead exposure.

Population characteristics such as density and the percentage of residents under 18 are collected from US Census data^42^. We use the Social Vulnerability Index (SVI) for 2022 to capture the underlying socioeconomic and demographic conditions that influence how communities are affected by lead exposure. SVI aggregates social factors into four categories: socioeconomic status, household composition, minority status, and housing/transportation^43^. Communities with a high social vulnerability index, characterized by low income, limited education, high minority populations, and inadequate access to transportation, may face greater barriers to identifying, reporting, and addressing lead exposure.

### Machine learning models

#### Logistic regression

Logistic Regression is one of the most widely accepted models for binary classification problems^44,45^. Logistic Regression is applied to determine the probability of occurrence when dependent variable is known^45^. Unlike linear regression, which predicts continuous values, logistic regression predicts the probability that a given input *x* belongs to a particular class^46^ (e.g., $y \in \{0,1\}$). The probability is modeled using the *logistic sigmoid function*, which maps real values to the interval [0, 1]:

1 $$\begin{aligned} P(y=1|x) = \sigma (z) = \frac{1}{1 + e^{-z}} \end{aligned}$$

where, z is represented as:

2 $$\begin{aligned} \quad z = \beta _0 + \sum _{j=1}^m \beta _j x_j \end{aligned}$$

where *z* is the linear combination of input features, $\beta _0$ is the intercept, and $\beta _j$ are the model coefficients for each feature $x_j$. The output $\sigma (z)$ represents the probability of the positive class.

#### Random forest

Random Forest is a robust ensemble learning algorithm that generates multiple decision trees and combines their predictions for classification and regression tasks. The algorithm creates bootstrap samples (random samples with replacement) from the original dataset $D = \{(x_i, y_i)\}$, where $x_i$ represents feature vectors and $y_i$ represents target classes. Then, Random Forest algorithm trains $T$ decision trees in parallel using these different samples. Random Forest selects a random subset of features at each node in every tree, for splitting, reducing correlation between trees and enhancing model diversity. For the final prediction, the algorithm aggregates outputs from all trees using majority voting for classification or averaging for regression:

3 $$\begin{aligned} \hat{y} = \text {Mode}\left( \{h_1(x), h_2(x), \dots , h_T(x)\}\right) , \end{aligned}$$

where $h_t(x)$ is the prediction of the $t$-th tree. Random Forest effectively mitigates overfitting by introducing randomness at both the data level (through bootstrap sampling) and feature level (through random feature selection). This enables the algorithm to excel with large datasets and maintain robust performance even with outliers and noise.

#### Adaptive boosting

Adaptive Boosting (AdaBoost) is the boosting machine learning technique that enhances classification accuracy by combining multiple weak learners into a strong classifier^47^. A key feature of AdaBoost is its ability to optimize the distribution of weights $w$ and train data $i$ in each iteration $t$ to effectively address unexpected outcomes. Initially, the AdaBoost algorithm selects a small subset of the training data $D = \{ (x_i, y_i) \}$, where each $x_i$ represents a vector of features from set $X$ and $y_i$ corresponds to the category from set $Y$. AdaBoost operates through sequential training iterations, where the algorithm adjusts sample weights adaptively based on previous classification results. This adaptation increases the importance of incorrectly classified examples by assigning them higher weights, making them more likely to be classified correctly in subsequent rounds. The algorithm weights each classifier’s contribution according to its performance, giving more influence to more accurate classifiers. This iterative process persists until reaching perfect classification of the training data or completing a predetermined number of iterations.

For each classifier training, the algorithm examines all possible functions and calculates the error for each. The optimal function is then identified as the first weak classifier. The initial learner’s task is to find a weak hypothesis $h_t: X \rightarrow \{-1, +1\}$ that is acceptable for the distribution $D_t$. The goal is to select $h_t$ to minimize the error $\epsilon _t$, defined as:

4 $$\begin{aligned} \epsilon _t = P_{i \sim d_t}(h_t(x_t) \ne y_i) \end{aligned}$$

Subsequently, the coefficient $\alpha _t$ is computed as:

5 $$\begin{aligned} \alpha _t = \frac{1}{2} \ln \left( \frac{1 - \epsilon _t}{\epsilon _t}\right) \end{aligned}$$

By adjusting the distribution $D$ and emphasizing the areas of misclassifications, the final AdaBoost classifier is expressed as:

6 $$\begin{aligned} H(x) = \text {Sign}\left( \sum _{t=1}^{T} \alpha _t h_t(x)\right) \end{aligned}$$

Here, $h_t$ represents the weak learner, $\alpha _t$ is the coefficient, and the outcome of the final hypothesis is denoted as $H(x)$. AdaBoost model operates by iteratively correcting mistakes from the weak learners. By combining multiple weak learners, AdaBoost reduces the bias of final prediction model^47^. We select AdaBoost model based on the merit of accuracy improvement, reducing bias and minimal hyperparameter tuning requirements^48,49^.

#### Extreme gradient boosting

Chen and Guestrin (2016) introduced the *eXtreme Gradient Boosting* (XGBoost) algorithm as an extension of gradient boosting (GB)^50^. XGBoost integrates multiple weak learning models (i.e., decision trees) to generate a strong learner^51,52^. Traditional boosting methods use the first derivative of the loss functions^50^. XGBoost advances similar procedure by applying a Taylor series expansion to better approximate the loss function.

The objective of XGBoost is to minimize a regularized objective function, expressed as:

7 $$\begin{aligned} L(\Phi ) = \sum _i l(\hat{y}_i, y_i) + \sum _k \Omega (f_k) \end{aligned}$$

The first term in Eq. (14) represents the *loss function*, which measures the error between the true label $y_i$ and the predicted label $\hat{y}_i$. The second term, $\Omega (f_k)$, acts as a *regularization component* to control model complexity and prevent overfitting, defined as:

8 $$\begin{aligned} \Omega (f) = \gamma T + \tfrac{1}{2} \lambda \Vert w \Vert ^2 \end{aligned}$$

Here, *T* is the number of leaves in the decision tree, *w* is the score assigned to each leaf, and $\gamma$ and $\lambda$ are regularization parameters. The learning process proceeds iteratively, where at each step *t*, the algorithm minimizes:

9 $$\begin{aligned} L^{(t)} = \sum _{i=1}^n l\Big (y_i, \hat{y}_i^{(t-1)} + f_t(x_i)\Big ) + \Omega (f_t) \end{aligned}$$

To further accelerate optimization, a second-order Taylor expansion of the loss function is applied, enabling faster and more efficient training. We select XGBoost based on the merit of unique objective function and flexibility in selecting loss functions^51^.

#### Light gradient boosting

Light Gradient Boosting (LightGBM) uses a *leaf-wise* growth approach where the leaf providing the largest reduction in the loss function is expanded first. This procedure makes the algorithm more efficient and achieves better accuracy. For the identification of the optimal split, LightGBM adopts histogram based approach, where feature values are discretized into bins, and histograms are constructed to determine the best split points. LightGBM updated only the leaf with the smallest number of data samples, as non-parent histograms can be generated by subtracting the parent histogram. The main flow of the LightGBM algorithm is given as:

10 $$\begin{aligned} F_n(x) = \alpha _0 f_0(x) + \alpha _1 f_1(x) + \cdots + \alpha _n f_n(x) \end{aligned}$$

where the classifier is initialized with *n* decision trees, and the weight of each training sample is $\tfrac{1}{n}$. The weak classifier *f*(*x*) is iteratively trained, with its weight $\alpha$ determined at each step. The algorithm continues updating the weights until the final classifier $F_n(x)$ is obtained.

We select Bayesian Optimization to fine-tune the hyperparameters of the Random Forest, Logistic Regression, XGBoost, LightGBM and AdaBoost classifiers. Bayesian Optimization efficiently explores the parameter space by using a probabilistic model to select the most promising hyperparameter combinations. First we optimize the minimum number of samples required to split an internal node (min_samples_leaf), the maximum depth of the tree (max_depth), and the minimum number of samples required to split an internal node (min_samples_split) during the construction of each decision tree^53^. Likewise, for the Logistic Regression model, we vary inverse of the regularization strength(C). We optimize rate at which the boosting algorithm learns from each iteration (learning_rate) for AdaBoost, XGBoost and LightGBM. Then, we vary the number of decision trees (n_estimators) in the all algorithms. Likewise, we use predict_proba function from sklearn library to determine the probability estimates.

#### Evaluation metrics

We use stratified 10-fold cross-validation to ensure robust and unbiased model evaluation^54^. We divide the entire dataset into ten equal-sized subsets while preserving the class distribution in each fold. For each iteration, we use nine folds training, and the remaining one for validation. We repeat the procedure ten times with each fold serving once as the validation set. By leveraging stratified cross-validation, we mitigate potential bias introduced by a single train-test split and ensure that the evaluation results generalize well across the entire dataset. We use the area under the Receiver Operating Characteristics (ROC) curve for assessing the predictive performance of model in each fold^55^. For each fold of cross-validation, we obtain the true alarm rate and false alarm rate. Since the ROC curves from different folds can vary in resolution, we apply linear interpolation to standardize the true alarm rates onto a common set of 100 equally spaced false alarm rates between 0 and 1. This ensures that all ROC curves are comparable across folds. Subsequently, we compute the mean true alarm rate at each false alarm rate by averaging the interpolated values from all folds. We estimate the standard deviation of true alarm rates at each point to quantify performance variability. We utilize these standard deviations to construct error bands representing ±1 standard deviation from the mean. In addition, predictive performance of both models is assessed using several standard classification metrics, including accuracy, precision, recall, and F1-score^56^:

11 $$\begin{aligned} \text {Accuracy}&= \frac{\text {TP} + \text {TN}}{\text {TP} + \text {TN} + \text {FP} + \text {FN}} \end{aligned}$$

12 $$\begin{aligned} \text {Recall}&= \frac{\text {TP}}{\text {TP} + \text {FN}} \end{aligned}$$

13 $$\begin{aligned} \text {Precision}&= \frac{\text {TP}}{\text {TP} + \text {FP}} \end{aligned}$$

14 $$\begin{aligned} \text {F1-score}&= 2 \times \frac{\text {Precision} \times \text {Recall}}{\text {Precision} + \text {Recall}} \end{aligned}$$

where,True Positive (TP) is the correctly identified lead hotspot, True Negative (TN) is the correctly identified non-lead location, False Positive (FP) is the location incorrectly classified as lead hotspot, False Negative (FN) is the location incorrectly classified as non-lead location. These metrics range from [0,1], where 0 indicates poor predictive performance and 1 indicates perfect predictive skill.

## Results

### Lead risk exposure

**Fig. 3:**
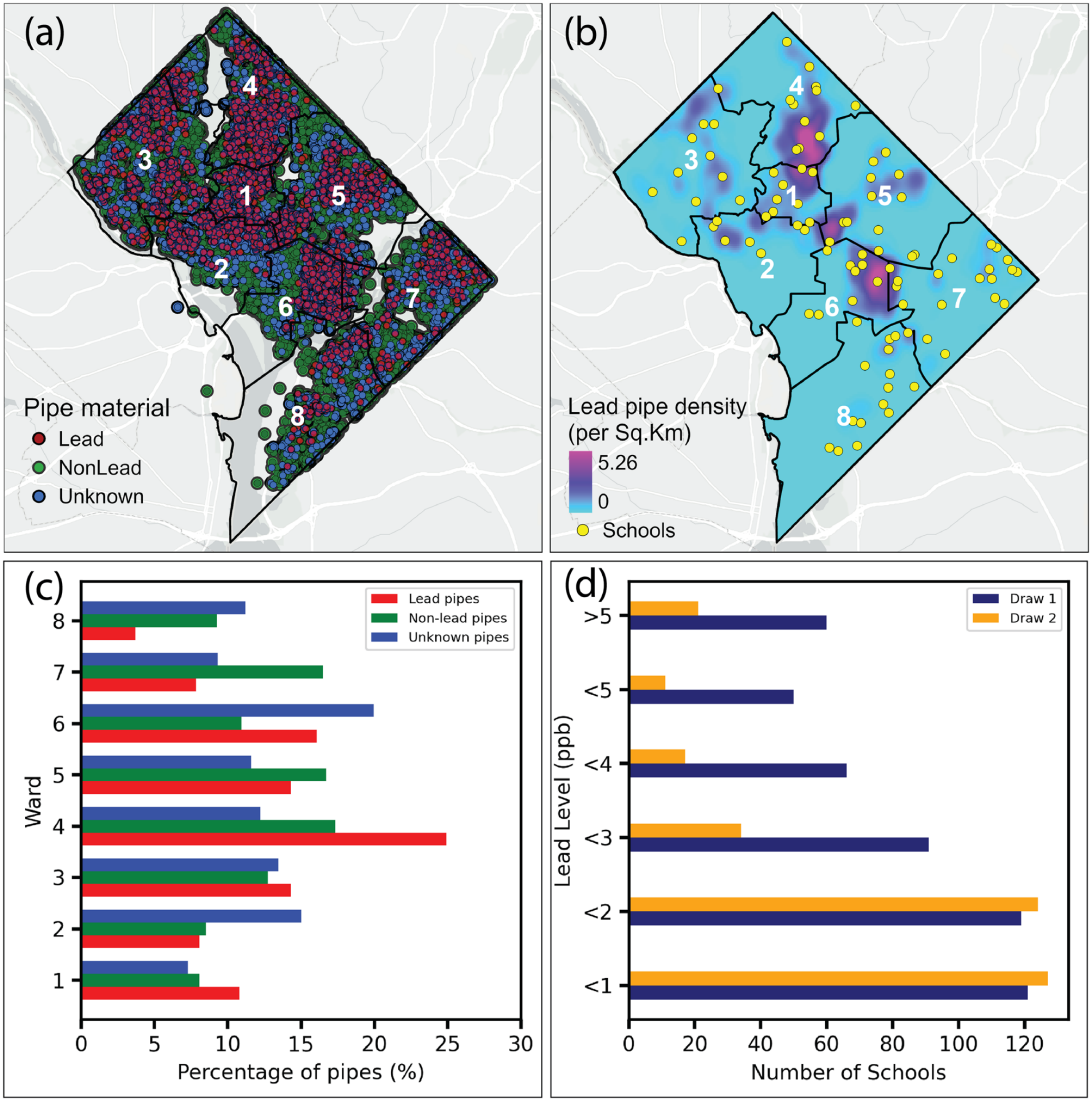
(**a**) Spatial distribution of the pipe materials in DC (DC Water, 2024); (**b**) lead pipe density and location of schools; (**c**) percentage of lead, non-lead and unknown pipes in each ward; (**d**) number of schools with different lead levels during lead test. Draw 1 represents the first drawn sample from the school tap. Draw 1 is a water sample collected after the water has been stagnant in the plumbing for a period, typically 8–18 hours. Draw 2 represents the second-drawn sample from the school tap. Draw 2 is collected after flushing the pipes to remove stagnant water. Basemap is obtained from ESRI worldmap V2^31^. Maps a and b are created using ArcGIS Pro^32^.

Figure 3 provides a spatial assessment of lead pipe distribution and associated lead exposure risks across DC. The spatial distribution of the service lines by material type reveals a widespread presence of lead pipes, with notable concentrations in Ward 1 (10. 8%), Ward 4 (24. 9%), Ward 5 (14.8 31%) and Ward 6 (16.06%) (Fig. 3a). High density of lead pipes shows multiple visually concentrated areas, some close to schools (Fig. 3b). The prevalence of pipes labeled ”unknown” further complicates accurate risk assessments and highlights gaps in infrastructure data. The unknown pipe ranges from 7.27% in Ward 1 to 19.9% in Ward 6.

School water sampling reveals that a substantial number of schools have detectable lead levels ($\ge$ 1 ppb) in both Draw 1 and Draw 2, with many exceeding critical safety thresholds of 5 ppb (Fig. 3d). Specifically, 60 schools in Draw 1 and 21 schools in Draw 2 recorded lead concentrations greater than 5 ppb, surpassing the American Academy of Pediatrics’ recommendation of 1 ppb^57^ (Fig. 3d). The sampling data shows that more than 100 schools reported detectable lead concentrations in Draw 1 below 1 ppb, while nearly 60 schools fall into higher concentration categories ($\ge$ 5 ppb). More concerning, 14 schools in Draw 1 and 9 schools in Draw 2 exceeded the US EPA’s action level of 15 ppb^10^. This underscores the persistence of contamination risks, as flushing between draws may offer only temporary mitigation and no level of lead in blood is considered safe.

### Drivers of lead contamination

**Fig. 4:**
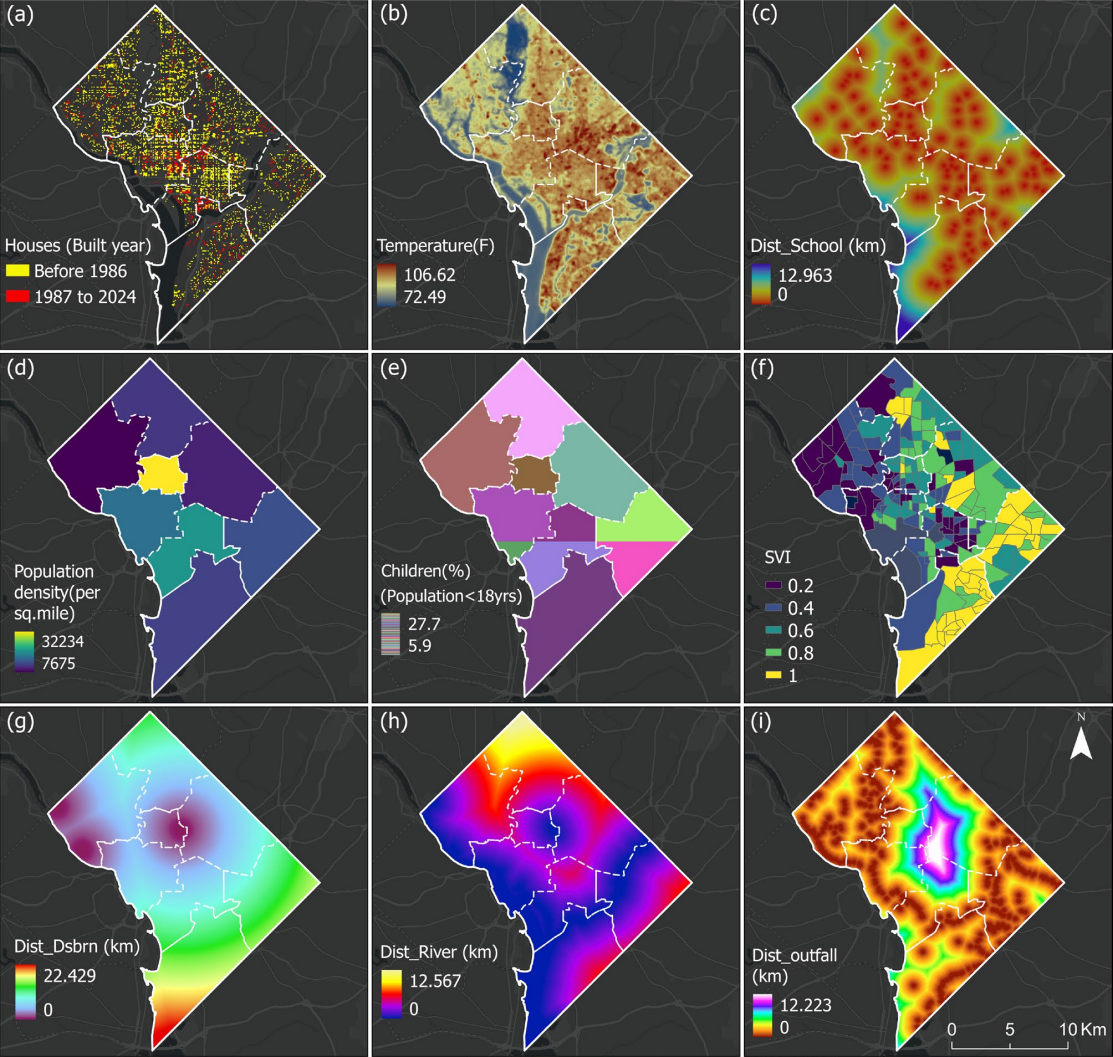
Input features for the machine learning models representing (**a**) spatial distribution of buildings constructed in DC; (**b**) average land surface temperature; (**c**) distance of each grid to the non-lead pipes (Dist_Nonlead) determined using Euclidean distance; (**d**) population density; (**e**) percentage of population having age below 18; (**f**) Social Vulnerability Index (SVI); (**g**) distance of each grid to the water reservoir before distribution (Dist_dsbrn); (**h**) distance of each grid to the nearest streams (Dist_river); and (**i**) distance of each grid to the combined stormwater outfall (Dist_outfall). Basemap is obtained from ESRI worldmap V2^31^. Map created using ArcGIS Pro^32^.

Figure 4 highlights key environmental and demographic drivers of lead contamination risk. Areas with a high concentration of older homes built before 1986, particularly in the central and northeastern parts of the city (Fig. 4a), are at greater risk due to the potential presence of lead-based paint and aging plumbing. Central DC shows high population density (Fig. 4d), and when combined with a significant proportion of children (Fig. 4e), it raises concern due to children’s vulnerability to lead poisoning. Moreover, central DC lies in close proximity to rivers, storm drains, and outfalls (Fig. 4g–i), indicating a higher likelihood of lead transport through water pathways, especially in areas with legacy infrastructure. These highly concentrated urban features often lead to elevated temperatures (Fig. 4b), partly due to the formation of urban heat islands, which are localized areas of heat retention and increased air temperatures in and around urban centers^58^. Rising temperatures can enhance lead mobility in water systems by accelerating pipe corrosion, destabilizing protective mineral scales, and altering water chemistry through increased microbial activity and other temperature-driven processes. The distribution of social vulnerability across DC wards reveals strong geographic disparities (Fig. 4f). Wards 7 and 8, located in the southeastern part of the District, exhibit SVI above 0.8. These areas are marked by systemic disadvantages, including higher poverty rates, limited healthcare access, and greater exposure to environmental and infrastructural risks^59^. Wards 2 and 3, particularly in the northwest, show the lowest SVI ranging from 0.2 to 0.4, reflecting relatively advantaged populations with better access to resources and infrastructure^59^. Intermediate levels of vulnerability are observed in Wards 1, 4, 5, and parts of Ward 6, highlighting the need for nuanced, ward-specific policy interventions. These disparities underscore the importance of equity-centered planning and policy interventions to ensure that resilience-building efforts effectively prioritize the needs of at-risk populations.

### Lead contamination risk prediction

**Fig. 5:**
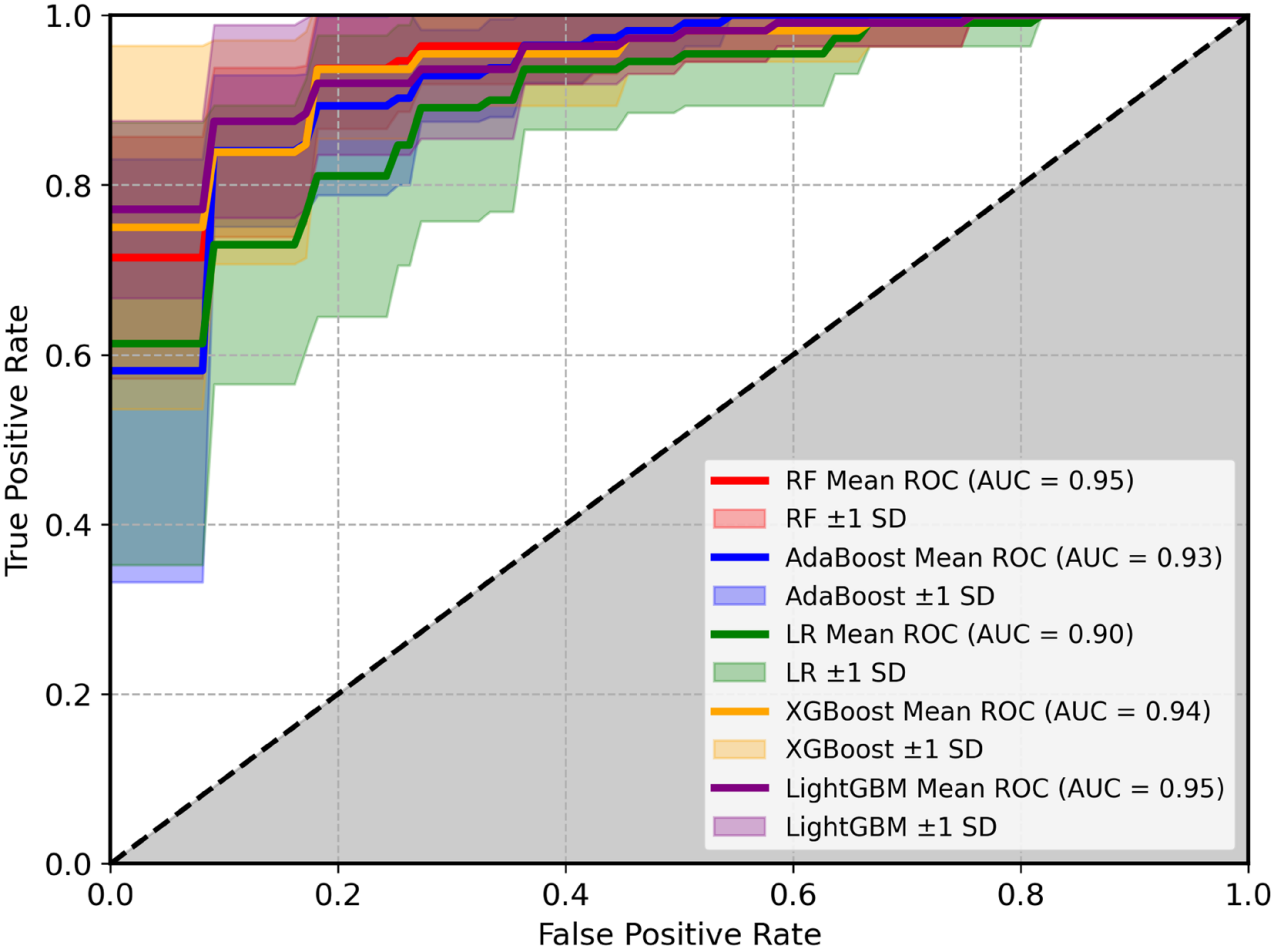
Performance assessment of Random Forest (RF), Adaptive Boosting (AdaBoost), Logistic Regression (LR), Extreme Boosting (XGBoost) and Light Gradient Boosting (LightGBM) using Receiver Operating Characteristic (ROC) curves for 10-fold cross-validation, with shaded regions around the ROC curve representing ±1 standard deviation.

With stratified 10-fold cross-validations, we determine the average area under the Receiver Operating Characteristic (ROC) curve (Fig. 5). The ROC curve assesses the quality of probability predictions by relating the probability of detection (true alarm) to the corresponding probability of false detection (false-alarm rate), as a decision threshold is varied across the full range of a continuous prediction quantity. The ROC analysis demonstrates strong predictive performance across all models, with AUC values ranging from 0.90 to 0.95. RF and LightGBM depict the highest discrimination ability (AUC=0.95, followed closely by XGBoost (AUC = 0.94) and AdaBoost (AUC = 0.93). The shaded regions around each curve represent ±1 standard deviation, indicating the stability of model predictions across folds. Ensemble-based methods not only outperformed Logistic Regression but also exhibited narrower confidence bands, reflecting greater robustness and consistency. In particular, Random Forest and LightGBM showed both high accuracy and low variance, making them the most reliable models for this application.

**Fig. 6:**
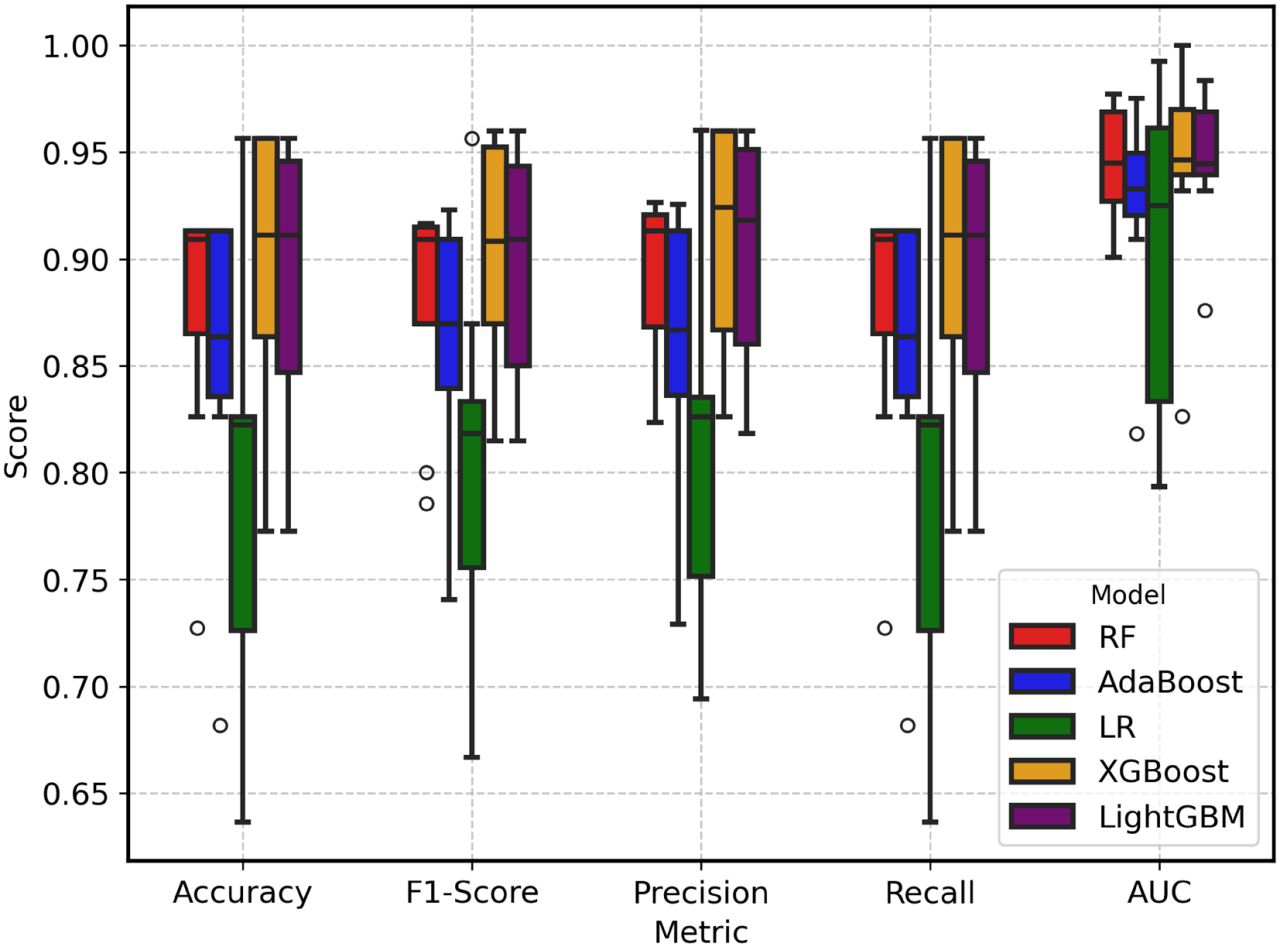
Performance assessment of Random Forest (RF), Adaptive Boosting (AdaBoost), Logistic Regression (LR), Extreme Boosting (XGBoost) and Light Gradient Boosting (LightGBM) using accuracy, F1-score, precision, recall, and area under the ROC curve (AUC).

We further evaluate the models by analyzing the distribution of classification metrics, including Accuracy, F1-Score, Precision and Recall (Fig. 6). Across all metrics, machine learning approaches demonstrated competitive performance, with median scores consistently above 0.85. Logistic Regression consistently exhibited the lowest predictive skill, with median scores around 0.80–0.85 across metrics, and demonstrated high variability, which indicates instability in its performance. In contrast, ensemble-based methods such as Random Forest, AdaBoost, XGBoost, and LightGBM achieved substantially higher medians, often above 0.90, with narrower interquartile ranges. XGBoost and LightGBM, in particular, achieved the highest median scores (0.95–0.97), though their performance exhibited slightly higher variance across folds, reflecting sensitivity to data partitioning. Random Forest emerged as a particularly attractive option. Its median performance, while marginally lower than XGBoost and LightGBM, was consistently competitive across all metrics. Importantly, Random Forest exhibited lower variance compared to boosting-based methods, which points to more stable and reliable performance. Compared to AdaBoost, RF achieved higher medians with comparable variability, underscoring its robustness.

**Fig. 7:**
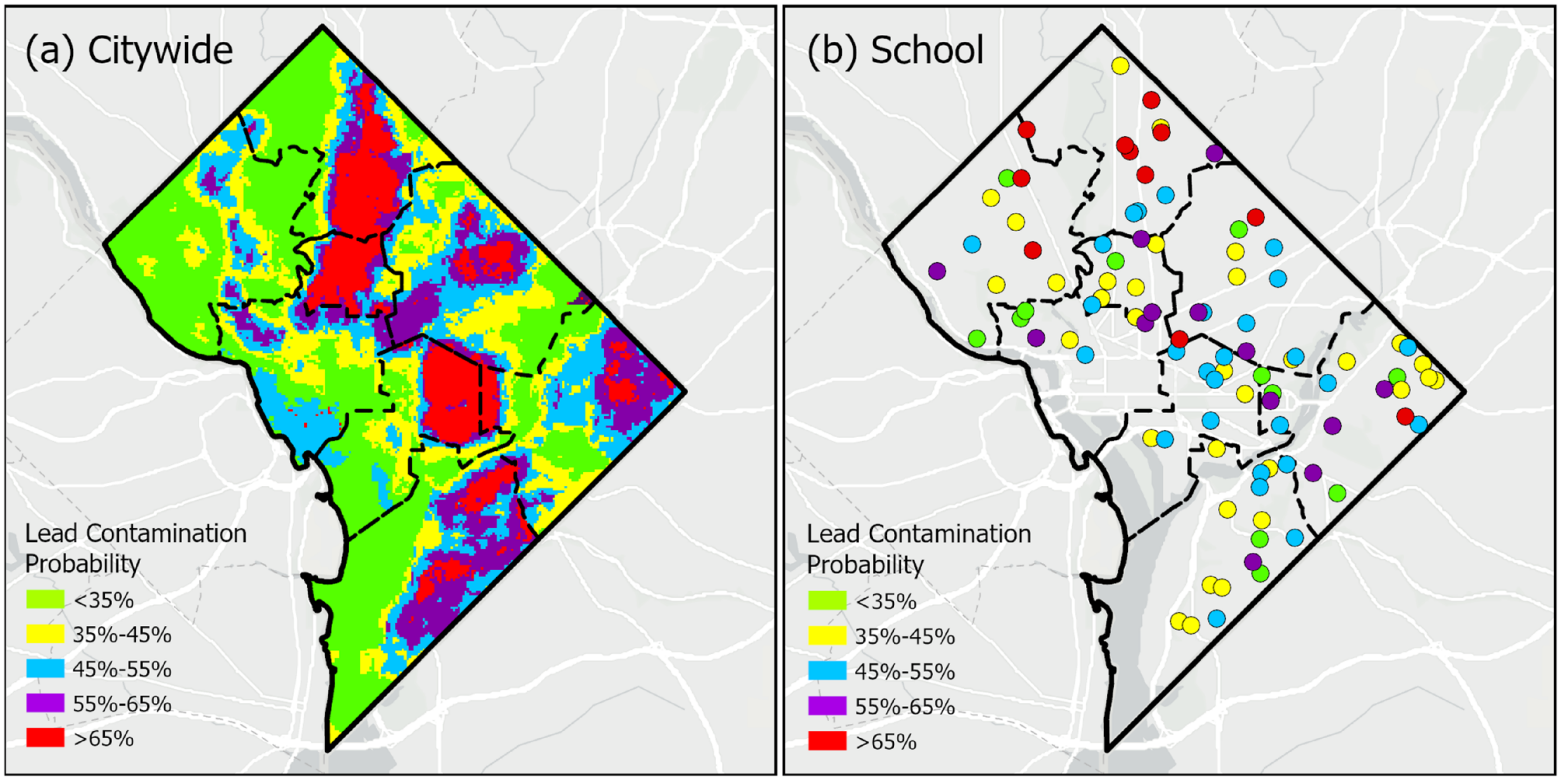
Prediction of lead contamination probability at (**a**) citywide and (**b**) school using Random Forest model.

We use Random Forest to predict the probability of lead contamination risk across wards (Fig. 7a) and schools (Fig. 7b). The maps employ a green-to-red color gradient representing a continuum from very low to very high contamination risk. To classify the predicted probabilities, we apply the natural breaks method and divide the data into five risk categories: very high, high, moderate, low and very low risk contamination zones. Our analysis shows that 11% of the total DC area lies into the very high-risk zone, 13.88% into high, and 38% into very low. The spatial distribution of contamination probability depicts distinct clusters (Fig. 7a). Very high-risk contamination zones are primarily concentrated in the central and northeastern parts of the city. Specifically, 10%, 18% and 11% of the high-risk areas are located within wards 1, 4 and 6, respectively. Moderate-risk zones are scattered throughout central and southeastern areas, while the northwestern and southwestern regions are dominated by lower-risk zones. These visible concentrated patterns emerge, in part, from infrastructure age cohorts sharing same construction periods(1880s-1950s) where heavy lead pipes are used. Localized hydrogeochemical conditions create distinct corrosion patterns. Conditions like pH levels, chloride concentrations, and dissolved oxygen vary spatially within distribution systems, accelerating lead dissolution in predictable geographic patterns. Buildings sharing the same water mains experience uniform flow dynamics, water age, and residual chemistry, producing consistent lead leaching rates within service zones. These mechanisms compound synergistically, as older housing clusters typically combine both legacy lead infrastructure and corrosive water conditions from aging distribution networks.

Figure 7b highlights the spatial variability of lead contamination risk across schools. Approximately 14% of schools are in high risk and 12% in very high risk zones. Schools located in Ward 4 exhibit particularly elevated risks. Many of these high-risk schools typically date back to the 1900 s and share similar demographic characteristics, construction periods, and local water conditions that promote lead corrosion. When compared to the citywide risk distribution (Fig. 7a), the school-level distribution reveals a notable alignment. Many of the schools at the highest risk are located within or adjacent to city regions identified as high or very high risk. This spatial overlap is especially evident in Wards 1 and 4, where both school-level and citywide analyses indicate high lead contamination probability. Our predictive approach can help identify high-risk schools where lead testing should be prioritized, allowing for more efficient allocation of limited resources for remediation efforts.

### Feature importance

**Fig. 8:**
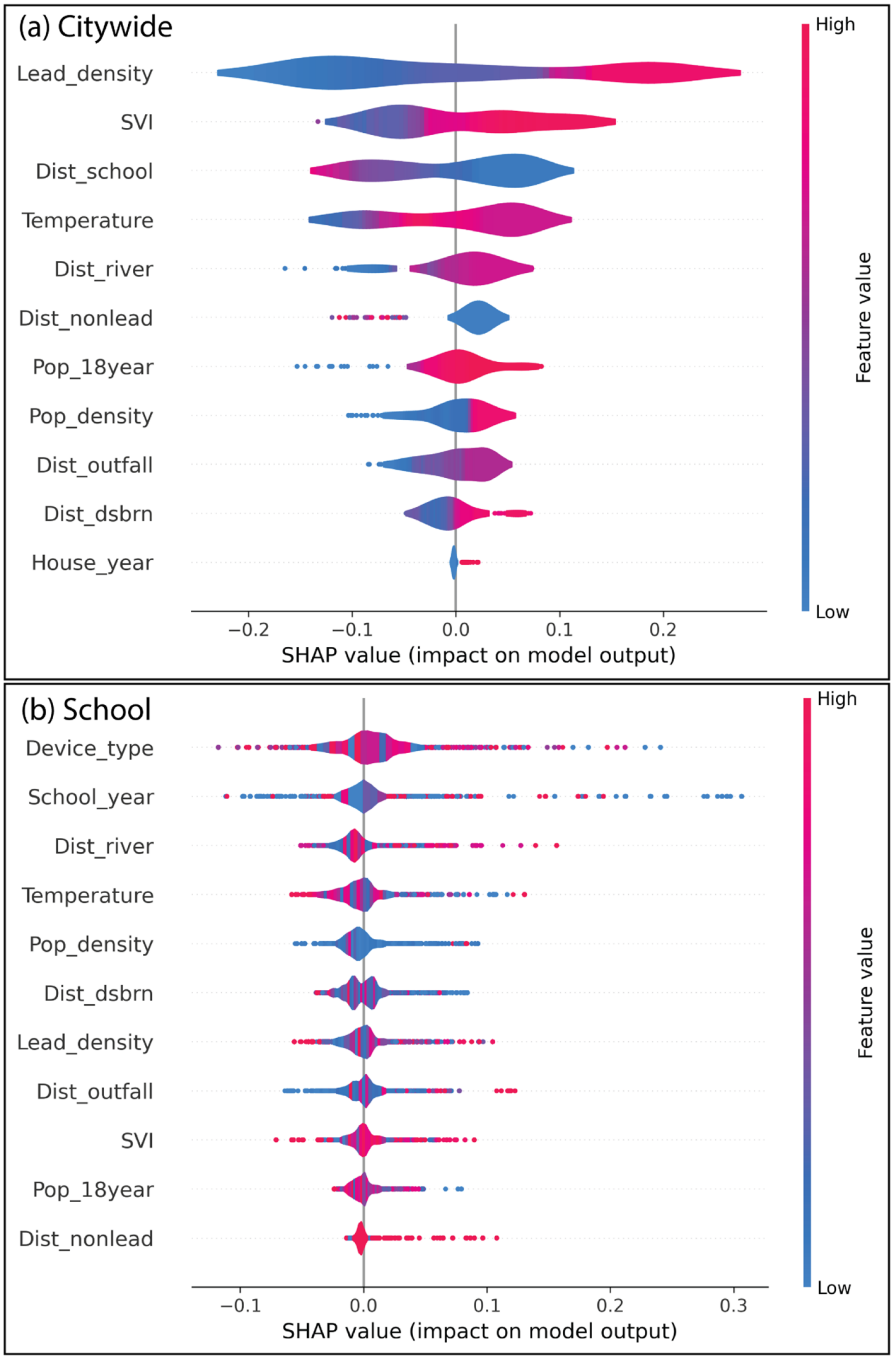
(**a**) Feature importance for (**a**) city-wide, and (**b**) school lead risk contamination predictions. Lead_density represents the distribution density of lead pipes; SVI represents Social Vulnerability Index; Dist_school represents distance of each grid to the school; Temperature represents average land surface temperature; Dist_river represents the distance of each grid to the nearest streams; Dist_Nonlead represents the distance of each grid to the non-lead pipes; Pop_18year represents percentage of population having age below 18 year; Pop_density represents population density; Dist_outfall represents distance of each grid to the combined stormwater outfall; Dist_dsbrn represents distance of each grid to the water reservoir before distribution; House_year represents the construction year of buildings in DC; Device_type represents the device from where water is collected in schools; and School_year represents the construction year of school in DC.

**Fig. 9:**
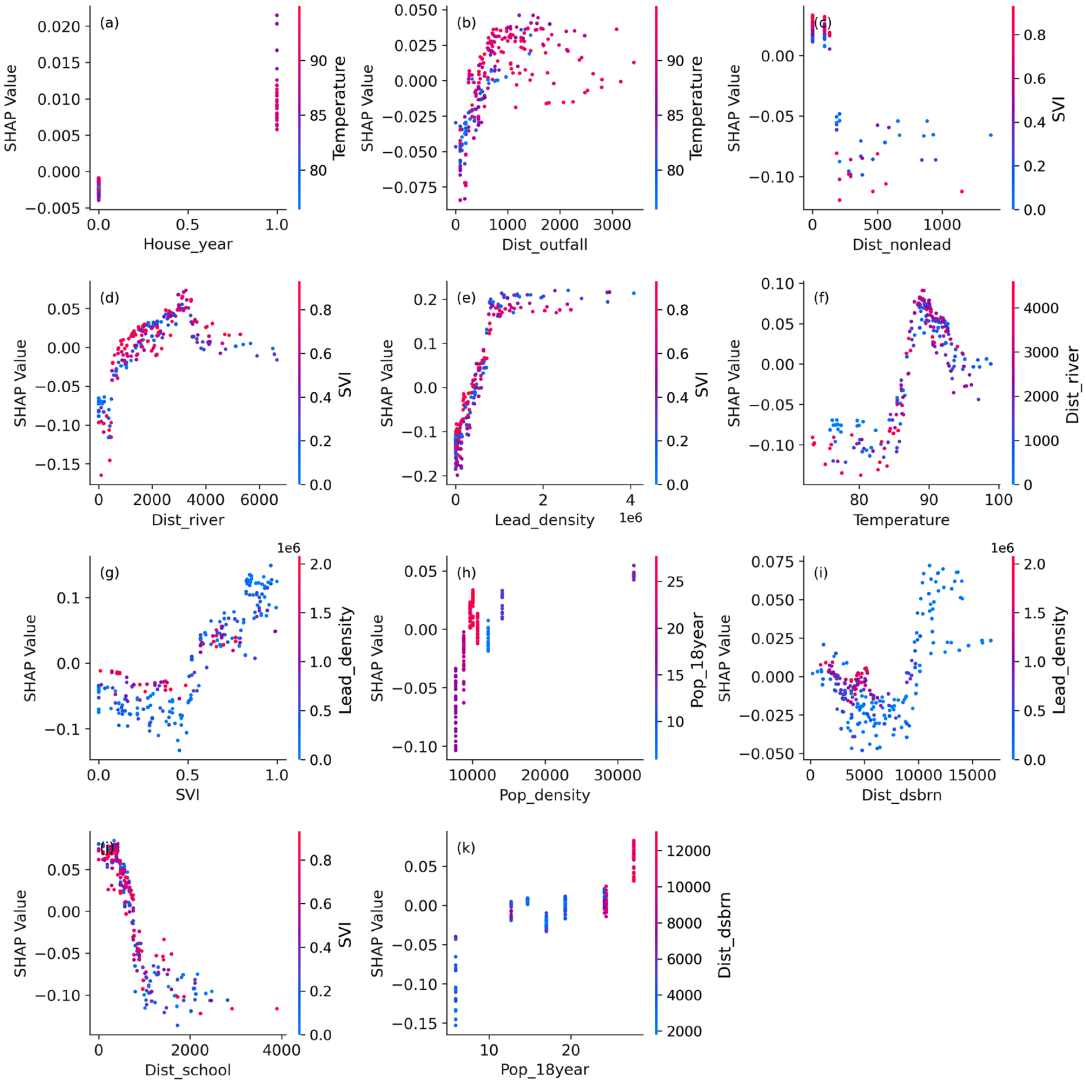
Dependence plot for for city-wide analysis. Lead_density represents the distribution density of lead pipes; SVI represents Social Vulnerability Index; Dist_school represents distance of each grid to the school; Temperature represents average land surface temperature; Dist_river represents the distance of each grid to the nearest streams; Dist_Nonlead represents the distance of each grid to the non-lead pipes; Pop_18year represents percentage of population having age below 18 year; Pop_density represents population density; Dist_outfall represents distance of each grid to the combined stormwater outfall; Dist_dsbrn represents distance of each grid to the water reservoir before distribution; House_year represents the construction year of buildings in DC.

**Fig. 10:**
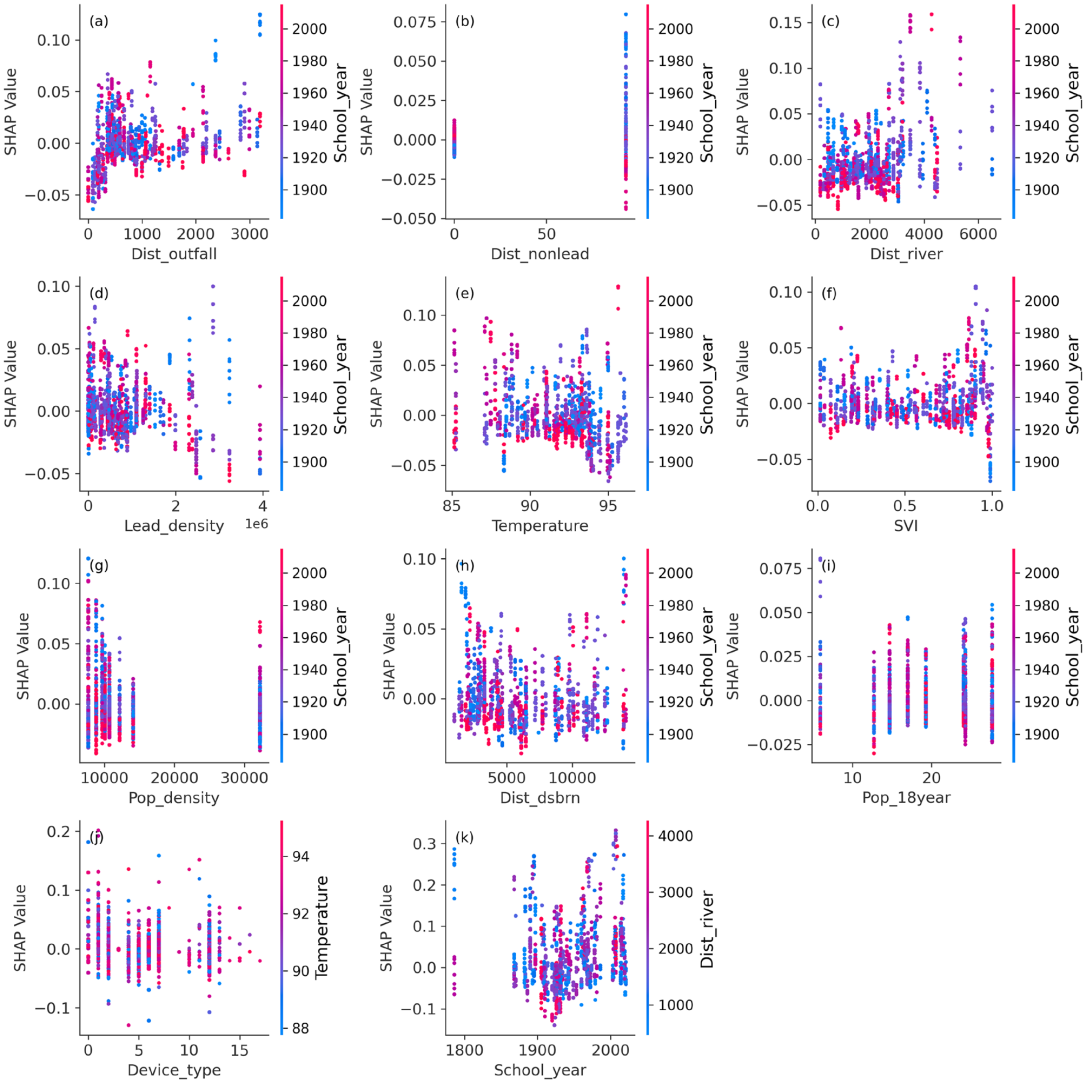
Dependence plot for school-based analysis. Lead_density represents the distribution density of lead pipes; SVI represents Social Vulnerability Index; Dist_school represents distance of each grid to the school; Temperature represents average land surface temperature; Dist_river represents the distance of each grid to the nearest streams; Dist_Nonlead represents the distance of each grid to the non-lead pipes; Pop_18year represents percentage of population having age below 18 year; Pop_density represents population density; Dist_outfall represents distance of each grid to the combined stormwater outfall; Dist_dsbrn represents distance of each grid to the water reservoir before distribution; House_year represents the construction year of buildings in DC; Device_type represents the device from where water is collected in schools; and School_year represents the construction year of school in DC.

SHapley Additive exPlanations (SHAP) quantifies the marginal impact of each feature by considering all possible combinations of inputs and distributing the prediction credit according to principles of cooperative game theory^60^. We employ SHAP to quantify both the importance of each feature and also indicate whether their contributions are positive or negative (Fig. 8). City-wide feature importance analysis indicates that environmental and infrastructure-related variables exert the strongest influence on model predictions of lead contamination risk (Fig. 8a). Among the input features, lead pipe density emerges as the most influential predictor across wards. This aligns with the city’s extensive legacy infrastructure, where many buildings are still serviced by lead pipes. We also use the SHAP dependence plot (Figs. 9 and 10) to understand how different features affect the model’s predictions. At lower lead density (Fig. 9e), the SHAP values exhibit a wider spread, indicating that lead density strongly impacts the prediction in both positive and negative directions. As lead density increases, the SHAP values become more constrained and show an upward trend, suggesting that higher lead density contributes positively to the model prediction.

SVI and proximity to schools substantially influence model performance. For SVI values below 0.5, the variable exerts a negative influence on the model prediction, while beyond this threshold, the SHAP values indicate a positive contribution (Fig. 9g). The areas with high SVI often have older housing with lead-based paint and plumbing, limited access to infrastructure upgrades, and closer proximity to industrial sources of lead. Socioeconomic barriers and reduced access to health resources in these areas further delay the detection and remediation of lead contamination, amplifying the impacts of exposure. Similarly, children spend significant time in school zones, requiring more frequent water testing. We find that shorter proximities to schools positively influence the model prediction, suggesting that closer access to schools is associated with higher predicted outcomes (Fig. 9j). Since the city-wide analysis incorporates children’s lead screening reports, proximity to schools affects the predictive outcomes.

Temperature and proximity to rivers also influence model performance, though to a slightly lesser degree. We observe a wider spread of SHAP values at the lower temperature and shorter proximities to rivers (Fig. 9d and f). As these values increase, the spread narrows and SHAP values mostly fall within the positive range. However, at extreme high temperatures and very close proximities to rivers, the spread of SHAP values widens again. Elevated temperatures can accelerate the corrosion of lead pipes and plumbing, thereby increasing lead leaching into drinking water. Proximity to rivers may be associated with higher lead concentrations, driven by industrial discharges, urban runoff, and sediment deposition. Moreover, riverine flooding can mobilize and redistribute contaminated sediments, intensifying exposure risks for nearby vulnerable communities. Other factors such as population density, distance to water distribution infrastructure, and housing construction year exert comparatively lower influence as evidenced by SHAP values clustered near zero (Fig. 8a), also supported by SHAP dependence plot (Fig. 9a, h and i).

The type of device used to collect water is the most influential predictor of lead contamination in schools (Fig. 8b). A wide variation in SHAP values is observed across different device types (Fig. 10j). This observation suggests that each device can exert both positive and negative influences on model performance. Devices such as water coolers and bottle fillers, particularly in older buildings, may contain lead solder or fittings, increasing the likelihood of lead leaching into stagnant water. This observation is reinforced by the school construction year emerging as the second most influential factor. We find upward trend in SHAP dependence plot for school construction year (Fig. 10k). Given that the use of lead in plumbing systems was banned in the US in 1986, schools built before this regulation are inherently more vulnerable to lead contamination. Proximity to rivers also ranks highly in importance, with schools located near waterways showing elevated predicted risks. SHAP dependence plot for proximity to rivers shows narrow spread in the lower range (Fig. 10c). In the school-focused analysis, a wider spread emerges at higher range, suggesting that schools located near waterways have higher tendency for lead contamination risks. This underscores the environmental dimension of lead exposure, particularly in areas prone to hydrological transport of contaminants. Temperature continues to play a significant role, as higher ambient temperatures are associated with increased lead solubility and, consequently, higher predicted exposure risks. While lead density remains an important feature, its relative influence diminishes in the school-specific model compared to the broader city-wide analysis, indicating a shift in dominant risk factors at the local scale.

## Discussion

Aging infrastructures have placed disproportionate burdens on historically underserved neighborhoods. Drinking water disparities stem not only from technical failures but also from social and institutional neglect^9^. Despite ongoing regulatory reforms and initiatives like Lead-Free DC^61^, lead exposure risks in DC remain unevenly distributed. These risks are primarily concentrated in neighborhoods with older housing, aging pipes, and high levels of social vulnerability. Similar patterns have emerged in several other US cities^62,63^. In Flint, Michigan, for instance, a combination of improper water treatment, aging pipes, and a delayed governmental response resulted in widespread lead exposure, disproportionately impacting low income and minority residents^23,62^. A similar situation occurred in Newark, New Jersey, where inadequate replacement of lead service lines, combined with high poverty rates and systemic disinvestment, contributed to prolonged exposure in predominantly Black and Latinx communities^63^. These examples highlight the structural inequities that continue to shape environmental health outcomes. Predictive modeling should target communities with aging infrastructure and high social vulnerability to identify emerging risks, guide timely interventions, and support more equitable infrastructure and public health strategies. To be most effective, predictive modeling must be grounded in locally relevant data, account for both environmental and social disparities, and be co-developed with community stakeholders to ensure that outputs are transparent, actionable, and aligned with community needs^64,65^.

In this study, we demonstrate the effectiveness of machine learning in lead risk prediction and underscore the critical role of explainable models to support decision-making in lead risk management. Machine learning methods, unlike traditional statistical approaches can process high-dimensional inputs and provide more accurate, localized risk assessments. Greater accuracy is often achieved through complex models; however, this typically comes at the expense of interpretability, making it difficult to discern how predictions are generated^27,66^. Such limitation in transparency and trust can hinder the broader adoption of machine learning in real-world decision-making^67^. Explainable machine learning can tackle these challenges by integrating diverse datasets, revealing complex nonlinear relationships, and providing transparent insights into model behavior.

This study highlights the critical intersection between lead contamination risks and environmental justice, emphasizing how social and institutional inequities exacerbate exposure in vulnerable communities. Disparities in infrastructure investment, historic disinvestment, and systemic neglect disproportionately affect low-income and minority neighborhoods, leading to uneven distribution of lead risk. Addressing these inequities requires not only technical solutions but also inclusive policy frameworks and community engagement to ensure that interventions are equitable and effective. Explainable machine learning models can help by providing transparent, actionable insights that can empower stakeholders and policymakers to prioritize resources and design interventions tailored to the unique needs of affected communities. Future efforts should focus on integrating social vulnerability metrics with environmental data and fostering partnerships with local communities to co-develop risk assessment and mitigation strategies that promote both environmental and social equity.

We recognize several limitations in this study. First, potential biases can result from incomplete or spatially uneven lead screening data. The use of a static temporal framework also restricts the model’s ability to account for dynamic changes, such as infrastructure upgrades, environmental remediation, or changing demographics. Additionally, discrepancies in spatial resolution between city- and school-level datasets may introduce uncertainty in localized predictions. Although the model incorporates key hydrological and environmental variables, it does not yet account for more complex processes such as groundwater flow, soil contamination, or urban runoff. Finally, the generalizability of the model to other urban settings has not been tested and warrants further investigation. Future work should aim to integrate temporal dynamics, include broader hydrological and environmental variables (e.g., soil lead, flooding risk), expand the framework to other cities to assess generalizability and quantify the uncertainty. These limitations suggest that while our findings are informative, caution is needed when generalizing results beyond the studied context, and further research is necessary to refine and validate the model. Data availability and quality can vary greatly across locations, which may affect model accuracy and reliability. Additionally, differences in lead regulations, water treatment practices, and community engagement levels can influence model performance and relevance. Therefore, adapting the model to new areas will require careful calibration with local data, ongoing validation, and collaboration with local stakeholders to ensure the predictions are accurate and actionable for protecting drinking water safety.

Classification results are subject to uncertainties that stem from incomplete service line inventories, uneven spatial coverage of school water sampling, and potential errors in socioeconomic or pipe material records. Washington D.C consists of a combination of older and new service lines. Service line inventory data in older municipal systems is often incomplete or outdated due to sparse and inconsistent historical records. Pipe material classifications rely on available utility records that may contain inaccuracies regarding actual in-ground infrastructures installed decades ago. This could result in underestimation of lead exposure risk. In addition, schools in DC are spatially clustered near Rock Creek Park, Capitol Hill, and neighborhoods east of the Anacostia River. Collection of samples from these locations exhibits heterogeneity that may create blind spots in risk characterization. Furthermore, census-derived socioeconomic indicators may not fully capture neighborhood-level variations in vulnerability, especially in rapidly changing communities where demographic transitions often outpace standard data collection cycles. These uncertainties can introduce both random and systematic errors in machine learning predictions. Our models can underestimate the true exposure risks in data-sparse areas while overestimating them in well-documented regions. Future assessments would benefit from targeted field validation studies, standardized sampling protocols across jurisdictions, and incorporation of probabilistic modeling approaches to better characterize and propagate these uncertainties through the risk assessment framework.

## Conclusion

This study demonstrates the ability of explainable machine learning models to predict the risk of lead contamination in both city-wide and school-specific contexts in Washington, DC. By integrating hydrological, environmental, topographic, socioeconomic, and infrastructure characteristics, the model identified critical hotspots, with more than 25 % of the city and schools falling into high- or very-high-risk zones. In particular, Wards 1, 4, and 6 are among the most affected areas, exhibiting high concentrations of lead service lines and a higher predicted contamination risk. Lead pipe density and social vulnerability are the most influential predictors at the city level, while school-level risks are primarily driven by infrastructure and environmental factors. We conclude that machine learning models hold significant potential to enhance lead risk prediction and support real-time decision-making in lead contamination risk management.

These findings can have important implications for city policymakers and infrastructure planners. By identifying high-risk areas and the key factors driving lead contamination, this study can help prioritize investments in lead pipe replacement and targeted public health interventions. Incorporating explainable machine learning models into routine risk assessment can improve transparency and build trust with affected communities. We recommend that future city policies integrate data-driven, equitable approaches to infrastructure upgrades and community engagement to effectively reduce lead exposure and promote environmental justice.

## Data Availability

A test case for generating lead hotspots prediction is available at the GitHub repository (lead). The full data-model configuration will be made available upon request to the corresponding author.
